# The Impact of Healthy Parenting As a Protective Factor for Posttraumatic Stress Disorder in Adulthood: A Case-Control Study

**DOI:** 10.1371/journal.pone.0087117

**Published:** 2014-01-29

**Authors:** Adriano R. Lima, Marcelo F. Mello, Sérgio B. Andreoli, Victor Fossaluza, Célia M. de Araújo, Andrea P. Jackowski, Rodrigo A. Bressan, Jair J. Mari

**Affiliations:** 1 Department of Psychiatry, São Paulo School of Medicine, Federal University of São Paulo (UNIFESP), São Paulo, Brazil; 2 Department of Mathematics and Statistics, University of São Paulo/USP, São Paulo, Brazil; 3 Interdisciplinary Laboratory of Clinical Neuroscience (LiNC), Federal University of São Paulo (UNIFESP), São Paulo, Brazil; University of Western Brittany, France

## Abstract

**Background:**

Early life social adversity can influence stress response mechanisms and is associated with anxious behaviour and reductions in callosal area later in life.

**Objective:**

To evaluate the association between perceptions of parental bonding in childhood/adolescence, hypothalamic-pituitary-adrenal (HPA) axis response, and callosal structural integrity in adult victims of severe urban violence with and without PTSD.

**Methods:**

Seventy-one individuals with PTSD and 62 without the disorder were assessed with the Parental Bonding Instrument (PBI). The prednisolone suppression test was administered to assess cortisol levels, and magnetic resonance imaging was used to assess the total area of the corpus callosum (CC), as well as the areas of callosal subregions.

**Results:**

The PBI items related to the perception of ‘not having a controlling mother’ (OR 4.84; 95%CI [2.26–10.3]; p = 0.01), ‘having a caring father’ (OR 2.46; 95'%CI [1.18–5.12]; p = 0.02), and ‘not having controlling parents’ (OR 2.70; 95%CI [1.10–6.63]; p = 0.04) were associated with a lower risk of PTSD. The PTSD group showed a blunted response to the prednisolone suppression test, with lower salivary cortisol levels upon waking up (p = 0.03). Individuals with PTSD had smaller total CC area than those without the disorder, but these differences were not statistically significant (e-value  = 0.34).

**Conclusions:**

Healthy parental bonding, characterized by the perception of low parental control and high affection, were associated with a lower risk of PTSD in adulthood, suggesting that emotional enrichment and the encouragement of autonomy are protective against PTSD in adulthood.

## Introduction

Investigations of the etiology of PTSD have revealed that nearly 30% of individuals exposed to traumatic events will develop, among other stress-related disorders, the disorder in its full form. The “incomplete penetrance” of the traumatic event suggests that other factors may also contribute to the development of PTSD [1], [2]. One such factor may be an innate, instinctive system of emotional and behavioral patterns, referred to by Bowlby as the *attachment system* [3]. The influence of parental bonding on genetic expression and modulation, affective regulation, mentalization and social interactions are at the core of investigations of gene-environment interactions and, consequently, of the repercussions of such interactions on development as a whole [4].

Many of the risk and resilience factors for PTSD are related to biological stress response mechanisms, which, in addition to being heritable, can be modulated by features of the child's early environment, such as parental bonding patterns [5]. Studies with animal models have demonstrated the importance of parental bonding for rodent development [6]; [7]; [8]. It has been verified that rats subjected to daily periods of maternal separation during the first two weeks of life presented persistently elevated corticotropin-releasing factor (CRF) mRNA levels not only in the hypothalamus, but also in the entire limbic system, including the amygdala. It is important to note that the long-term effects of maternal separation are influenced by factors such as the duration of the separation period and maternal behavior following separation from pups. In a study by Plotsky and Meaney [6], rat pups 2–14 days old were placed in one of three conditions: handling (15 min of separation from mother and home cage on a daily basis), maternal separation (MS; 180 min of comparable separation on a daily basis), or were left entirely undisturbed (non-handled; NH). Measurements of CRF mRNA levels in adult rats revealed that these values were higher in MS than in NH rats, and lower in H rats than in animals in either the MS or NH conditions. The same results were found in measurements of hypothalamic CRF levels under basal conditions. Significant CRF depletion was observed in rats when subjected to a 20-min period of restraint stress. However, the magnitude of the depletion was significantly lower in rats in the H condition than in those in the MS or NH conditions.

Paradoxically, impairments in HPA negative feedback inhibition and subsequent hypercortisolemia, which have been described in studies of major depression [9], [10], have not been observed in PTSD patients. However, both hypocortisolemia and hypocortisoluria are often found in PTSD patients [11]. Studies have shown that adult PTSD may also reflect the continuation of a response to an earlier exposure to an adverse situation, and that this process could be responsible for at least some of the PTSD-associated changes in glucocorticoid responsiveness, and subsequent alterations in HPA axis response [12].

Studies of the associations between parental bonding, early exposure to adverse events and neural development have also shown a correlation between parental neglect and reductions in the area of the corpus callosum (CC) [13]. The presence of emotional neglect has been associated with reductions of 15 to 18% in the area of the anterior midbody and posterior regions of the CC. Similarly, the medial and posterior regions of the CC of individuals with a history of maltreatment in childhood have also been found to be smaller than the corresponding areas in control patients who had no early exposure to adverse events [14].

In the face of these findings, which inter-relate parental bonding, neuroendocrine response to stress, and brain development, the intent of the present study was to establish possible associations between parental bonding perceptions, hypothalamic-pituitary-adrenal (HPA) axis response, callosal integrity, and the development of PTSD in adulthood. The main aim of the study was to test whether healthy parental relations, characterized by emotional enrichment and low parental control, are a protective factor against the development of PTSD in adulthood.

## Materials and Methods

### Subjects

The study was approved by the Ethics Committee of the Federal University of São Paulo (UNIFESP), Brazil (protocol number 1169/10), and all participants provided written informed consent.

Eligible subjects were recruited from a large epidemiological survey conducted with a civilian population in the city of Sao Paulo, aged 18 to 60 years. The survey measured life time trauma exposure in the community, and individuals were referred to a trauma out-patient clinic from the Federal University of São Paulo, for further assessment. [15], [16]. All participants were initially assessed with the *Structured Clinical Interviews for DSM-IV Axis I and Axis II* (SCID-I and SCID-II, respectively), which were administered by a trained psychiatrist [17], [18]. Patients were included upon meeting DSM-IV diagnostic criteria for PTSD. The following exclusion criteria were applied: a) meeting DSM-IV criteria for borderline personality disorder, bipolar disorder, generalized anxiety disorder, panic disorder, obsessive compulsive disorder, major depressive disorder or substance dependence over the past six months; b) presence of acute or chronic diseases of the HPA axis (i.e.: Cushing or Addison disease); c) recent use (last six months) of psychotropic drugs or medications which would alter HPA axis response; and d) inability to understand the terms of the informed consent form. Participants were classified as PTSD+ and controls as PTSD- based on SCID results.

### Study Design and Procedures

The study used a case-control design. All participants took part in assessments of their perception of the quality of their parental relationship, during childhood and adolescence. The area of the CC was assessed using Magnetic Resonance Imaging (MRI). The *Parental Bonding Instrument (PBI)* was used to investigate parental behavior and healthy parent-child bonds [19]. The PBI is a 25-item instrument, derived from the factor analysis of 114 items drawn from the literature on parental qualities for adequate childhood and adolescent development. The PBI is a self-report, Likert type (0 to 3) instrument, with questions aimed specifically at the father and the mother, that measures two constructs, affect and control, for each parent, through which the individual is asked to evaluate parental behavior, during the first 16 years of their lives. The instrument has been translated and adapted to the Brazilian social and cultural context [20]. The HPA response to a pharmacological challenge was evaluated by comparing the cortisol concentrations in salivary samples collected before and after a prednisolone suppression test. Prednisolone was chosen, rather than dexamethasone, due to its similarity to endogenous cortisol and its ability to penetrate the blood-brain barrier, mimicking the response of the central nervous system to physiological adaptations to stress [21]. Prior to collecting biological samples, urine tests were carried out to detect illicit drug use and pregnancy. All participants were instructed to ingest one 5 mg prednisolone capsule at 10:00 PM, the night before a salivary sample was taken, and were told not to consume alcohol, coffee, tea or food after taking the pill. Cortisol secretion was assessed using Salivettes® upon waking, 30 min later, at 12:00 PM, 4:00 PM, 6:00 PM and 8:00 PM. The Salivettes® kits brought back by the subjects were prepared and sent to a laboratory for further analysis. After samples were collected from all subjects, cortisol levels in the saliva were assessed using the ELISA method. All samples were assayed in triplicate, using a Salimetrics cortisol kit (Salimetrics, State College PA, USA). Samples were analyzed at the “Genese Produtos Diagnósticos Ltd.” laboratory, and all data were entered into a database.

Imaging data were acquired at the “Instituto do Sono”, using a GE 1.5T Signa scanner, after cleaning for the standard exclusion criteria for the MRI procedure. Structural MR images were acquired using a sagittal T1 series (TR = 9.8 ms, TE = 3.1 ms, flip angle  = 30°, NEX = 1, matrix size  = 256×256, FOV = 24 cm, thickness  = 1.0 mm) yielding 124 slices. The DICOM files (T1-weighted images) were first converted to ANALYZE format using MRIcro. T1 images were then aligned along the midline of the brain and to the AC-PC axis using ANALYZE AVW 7.0. The mid-sagittal slice was identified based on the following neuroanatomical criteria: a) clear view of fornix and anterior commissure; b) clearly delineated cerebral aqueduct (Sylvian aqueduct); c) clearly delineated fourth ventricle; and d) clearly delineated CC. The mid-sagittal slice was then taken from the 3D volumetric data, and the CC was segmented into the seven subregions, outlined by Witelson, using the semi-automated Yale University in-house software. The seven callosal regions correspond to: 1) rostrum; 2) genu portion; 3) rostrum body; 4) anterior medial body; 5) posterior medial body; 6) isthmus; and 7) splenium. To adjust for brain size, the total brain volume was measured using the VBM Toolbox (dbm.neuro.uni-jena.of/vbm) [22] in SPM5 (Wellcome Department of Imaging Neuroscience, London, UK; www.fil.ion.ucl.ac.uk/spm). The sum of all voxel values within the segmented image approximates the total volume of the corresponding section. Total brain volume was calculated by adding the values obtained for gray and white matter volume. An inter-rater reliability of 0.99 (*p*<0.001) was obtained by comparing independent estimates of total callosal area and of CC subregions for ten patients. Intra-rater reliability was also calculated based on the same ten measurements of total CC area and of its seven subregions. The intra-rater reliability, for the area of subregions 1 through 7, were the following: 0.90 (P<0.001) for subregion 1, 0.94 (P<0.001) for subregion 2, 0.91 (P<0.001) for subregion 3, 0.95 (P<0.001) for subregion 4, 0.98 (P<0.001) for subregion 5, and 0.99 (P<0.001) for subregions 6 and 7. All reliability analyses were conducted using two-tailed Pearson correlation coefficients.

Evaluations of the neuroendocrine response to HPA axis suppression and structural analyses of the corpus callosum were only conducted for participants who also completed the assessment of the quality of the parental relationship, during childhood/adolescence.

### Statistical analysis and Data Presentation

Descriptive analyses were performed on all demographic and clinical variables. Means and standard deviations were computed for ordinal and continuous variables, and absolute and relative frequencies were calculated for categorical variables. Conclusions and generalizations were based on inferential analysis of the data. Between-group comparisons of categorical variables were carried out by means of Chi-square tests and odds ratios (OR) (95% confidence intervals). When the frequency in a cell was lower than five, the Chi-square Monte Carlo method was used. Continuous variables were compared between groups, using univariate analyses by means of the Mann Whitney non-parametric test. The Mann-Whitney non-parametric test was used in order to compare three or more groups. In situations where individuals were assessed more than once (i.e., cortisol levels over time, CC measurements), comparisons between groups were carried out by “non-parametric” repeated measures analyses for ordinal data [23]. Given the interdependence between variables and assuming a normal multivariate distribution, between-group comparisons of callosal data were carried out using the *FBST – Full Bayesian Significance Test* [24].

## Results

### Sample

The sample selected for this study was composed of 166 individuals (91 cases and 75 controls). Mean participant age was 39.6 years for cases (SE = 1.58) and 36.2 years for controls (SE = 1.05; p = 0.18). Out of the 91 individuals with a diagnosis of PTSD, only 71 were administered the PBI, as 19 could not be located and 1 refused to take part in the study. Of these 71 participants, 20 individuals underwent the evaluation of cortisol levels, 28 underwent a brain MRI scan and 12 took part in both evaluations. Out of the 75 individuals without the diagnosis of PTSD, 12 were not located and 1 refused to take part in the study, making for a total of 62 participants who were eligible for assessment with the PBI in this group. Of these 62 participants, who were eligible for assessment with the PBI, 12 had undergone the evaluation of cortisol levels, 21 had a brain MRI scan and 3 were assessed by both procedures. With respect to the sample loss, although it has been found differences between groups (PTSD+/PTSD−), these were not statistically significant, either for PBI scores (mothers: χ^2^ = 0.49; p = 0.48/fathers: χ^2^ = 1.26; p = 0.26), or for CC (χ^2^ = 3.10; p = 0.07) and cortisol measures (χ^2^ = 3.75; p = 0.05).

**Figure 1:**
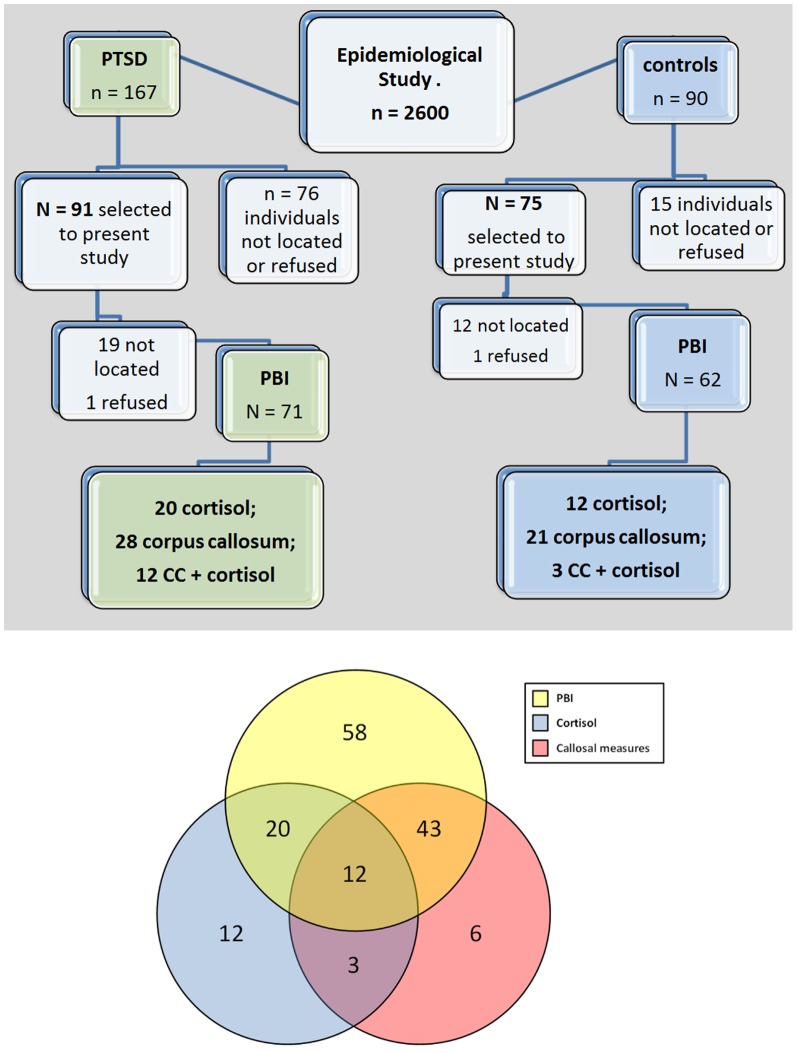
Summary. Flow chart of individuals throughout the study and Venn diagram to show the overlap between the subjects that completed each of the measures (PBI, cortisol, and callosal measures).

Figure 1 shows the flow chart of participants throughout the present study.

There were no significant between-group differences in the distribution of participant demographic characteristics, such as gender (χ2 = 0.69; p = 0.41), age (W = 2065.5; p = 0.18), marital status (χ2 = 2.22; p = 0.62), place of birth (χ2 = 0.05; p = 0.85), and schooling (χ2 = 0.57; p = 0.91) (Table 1).

**Table 1:** Demographic characteristics of the sample (n = 133) divided into PTSD cases (n = 71) and control subjects (n = 62).

		PTSD		Control		Total			
		freq	%	freq	%	freq	%	p-value*	Chi2
**Gender**	Female	53	74.6%	39	62.9%	92	69.2%	0.2023	16.256
	Male	18	25.4%	23	37.1%	41	30.8%		
**Marital Status**	Married	34	49.3%	37	59.7%	71	54.2%	0.5124	26.409
	Single	27	39.1%	16	25.8%	43	32.8%		
	Divorced	7	10.1%	8	12.9%	15	11.5%		
	Widowed	1	1.4%	1	1.6%	2	1.5%		
**Place of Birth**	São Paulo	49	72.1%	45	73.8%	94	72.9%	0.8463	0.0477
	Other	19	27.9%	16	26.2%	35	27.1%		
**Educational Level**	Illiterate	3	4.4%	5	8.3%	8	6.3%	0.7158	13.477
	Primary School	11	16.2%	11	18.3%	22	17.2%		
	High School	39	57.4%	34	56.7%	73	57.0%		
	College or Higher	15	22.1%	10	16.7%	25	19.5%		

Age of the sample (n = 133) divided into PTSD cases (n = 71) and control subjects (n = 62).								
					C. I			
Group	N	Mean	SD	SE	Lower Bound	Upper Bound	p-value	Statistic
**PTSD**	71	36.22	8.99	1.07	34.13	38.31	0,2096 ^1^	w = 1867
**Control**	62	39.63	13.00	1.65	36.39	42.86	0,0868 ^2^	t = −1,7285
**Total**	133	37.83	11.16	0.97	35.94	39.73		

p-value*: Chi Square Test; p-value^1^: Mann-Whitney Non-Parametric Test; p-value^2^: T-test; CI: Confidence Interval.

### Perceptions of parental bonding

From an initial sample of 166 potential subjects (91 cases and 75 controls), a total of 133 individuals met inclusion criteria for the study (71 cases and 62 controls). Among these 133 individuals, a total of 20 cases and 13 control patients were lost, between screening and study participation, due to the inability to locate individuals (19 cases and 12 controls) or refusal to take part in the study (1 case and 1 control).

#### Parents analyzed separately

The percentage of mothers rated as displaying high and low care on the PBI were 43.7% and 56.3%, respectively, in the PTSD+ group, and 46.8% and 53.2%, respectively, in the PTSD- group (p = 0.85). A total of 78.9%, and 21.1% of mothers were rated, respectively, as having high and low control in the PTSD+ group, while 43.5% and 56.5% of mothers were described as having high and low control by PTSD- participants (p<0.01). The percentage of fathers rated as displaying high and low care were 34.9% and 65.1%, respectively, in the PTSD+ group, and 56.9% and 43.1% in the PTSD-group (p = 0.02), while ratings for high and low overprotection were, respectively, 71.4% and 28.6% for the PTSD+ group, and 63.8% and 36.2% for the PTSD- group(p = 0.48). Low control by mothers (OR 4.84; 95%CI [2.26–10.3]; p = 0.01) and high care by fathers (OR 2.46; 95%CI [1.18–5.12]; p = 0.02) were significantly more frequent in the PTSD- group (Table 2).

**Table 2:** Distribution of PBI care and control scores for the mothers and fathers of patients with PTSD and controls.

		PTSD		Control		Total			
		N	%	N	%	N	%	OR [CI]	p-value
**PBI Affect Mother**	**Low**	40	56.3%	33	53.2%	73	54.9%	1.13	0.85
	**High**	31	43.7%	29	46.8%	60	45.1%	[0.57;2.25]	
**PBI Control Mother**	**High**	56	78.9%	27	43.5%	83	62.4%	4.84	0.01*
	**Low**	15	21.1%	35	56.5%	50	37.6%	[2.26;10.3]	
**PBI Affect Father**	**Low**	41	65.1%	25	43.1%	66	54.5%	2.46	0.02*
	**High**	22	34.9%	33	56.9%	55	45.5%	[1.18;5.12]	
**PBI Control Father**	**High**	45	71.4%	37	63.8%	82	67.8%	1.42	0.48
	**Low**	18	28.6%	21	36.2%	39	32.2%	[0.66;3.05]	

PBI: Parental bonding instrument; p-value: Chi Square Test;

* statistically significant; OR: Odds Ratio; CI: Confidence Interval.

#### Analysis for combined parent scores

The percentage of parents rated as showing high and low care on the PBI were, respectively, 27.0% and73.0% in the PTSD group, and 39.7% and 60.3% in the control group (p = 0.19). Parents were classified as having high and low control by 85.7% and 14.3% of the PTSD group, and 69.0% and 31.0% of the control group, respectively (p = 0.04). Low control was significantly more frequent in the PTSD- group (OR 2.70; 95%CI [1.10–6.63]; p = 0.04) (Table 3). When analyses were performed on the bonding categories, which group care and control scores, healthy parental bonds – *optimal parenting* – were more frequent in the group without PTSD, but this difference was not statistically significant (OR 2.79; 95%CI [1.05–7.44]; p = 0.06) (Table 3).

**Table 3:** The distribution of mothers and fathers of patients with PTSD and controls into the main bonding categories – *optimal x dysfunctional parenting* – defined by the PBI.

		PTSD		Control		Total		OR [CI]	p-value
		N	%	N	%	N	%		
**PBI Affect Parents**	**Or Mother or Father Low**	46	73.0%	35	60.3%	81	66.9%	1.78	0.198
	**Both Parents High**	17	27.0%	23	39.7%	40	33.1%	[0.83;3.82]	
**PBI Control Parents**	**Or Mother or Father High**	54	85.7%	40	69.0%	94	77.7%	2.70	0.046*
	**Both Parents Low**	9	14.3%	18	31.0%	27	22.3%	[1.10;6.63]	
**PBI Parents**	**Others**	56	88.9%	43	74.1%	99	81.8%	2.79	0.062
	**BothOptimal Parenting**	7	11.1%	15	25.9%	22	18.2%	[1.05;7.44]	

PBI: Parental bonding instrument; p-value: Chi Square Test;

* statistically significant; OR: Odds Ratio; CI: Confidence Interval.

### Callosal measures

**Figure 2:**
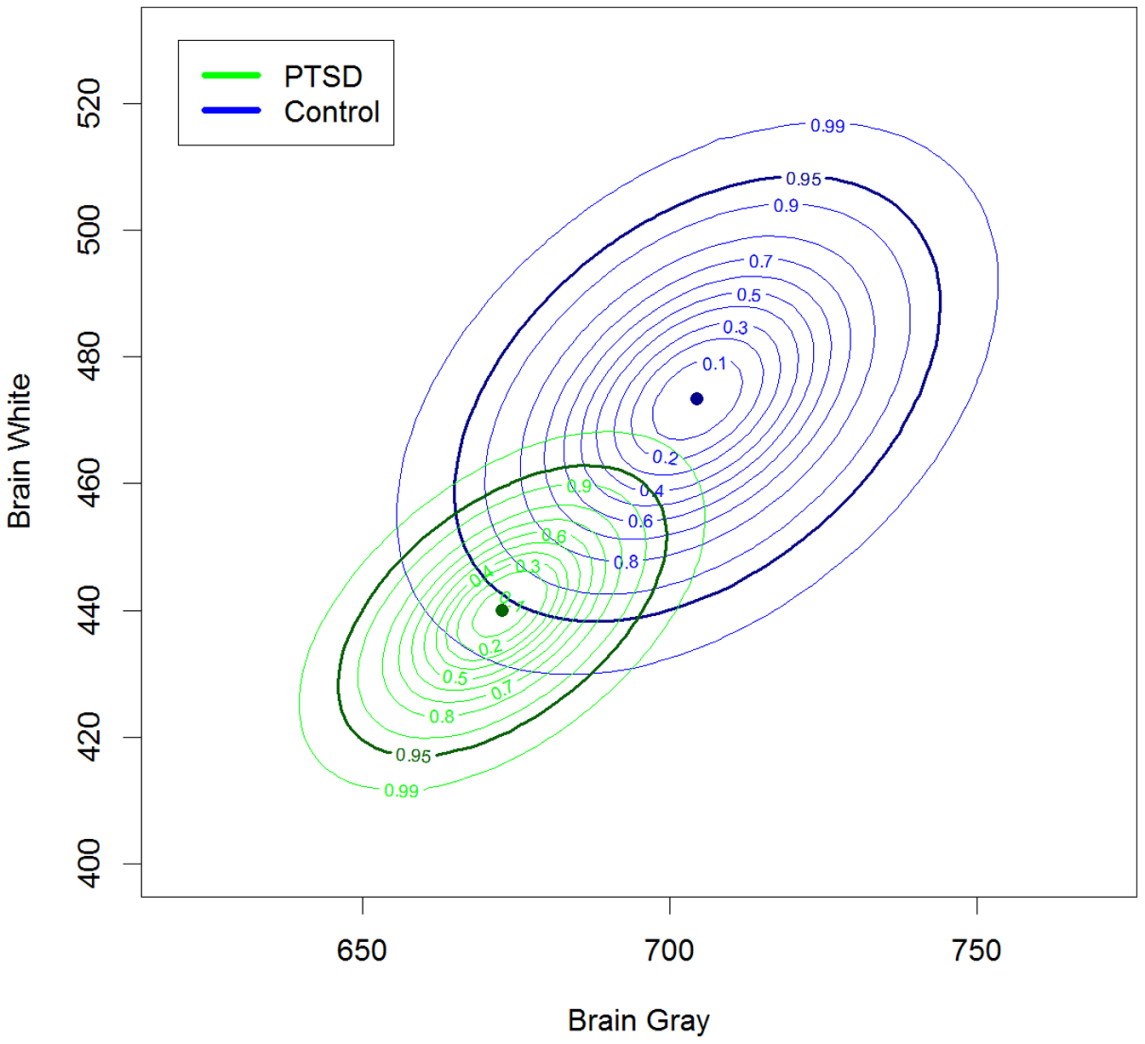
Corpus callosum. Level curves of the *a posteriori* distribution of mean white matter and gray matter area in patients with PTSD and controls. The darker curve represents the 95% confidence interval.

One hundred and nineteen individuals were initially selected for the study, but only 64 subjects (40 PTSD and 24 controls) met the inclusion criteria for a brain MRI scan. Among the individuals who were excluded of the CC neuroimaging assessment, 46.2% had a diagnosis of PTSD, 67.9% were female, 51.3% were married, 71.8% were born in São Paulo, whose average age was of 38.04 (SE = 1.35). On the other hand, for those who were included in CC neuroimaging, 63.6% had a diagnosis of PTSD, 70.9% were female, 56.4% were married, 67.7% were born in São Paulo, whose average age was 37.55 (SE = 1.39). There were no statistical differences between groups. The mean gray matter area in the groups with and without PTSD were, respectively, 672.67 cm2 (SE = 10.91) and 704.45 cm2 (SE = 16.14), while mean white matter area was, 439.92 cm2 (SE = 9.34) in the PTSD+ group, and 473.39 cm2 (SE = 14.35) in the PTSD- group. Figure 2 displays the *a posteriori* distribution curves for the data, in which the darker curve represents the 95% confidence interval. As shown by the overlapping intervals, although there were differences between the groups - individuals with PTSD had smaller total CC area - these differences were not statistically significant (e-value  = 0.34) (Figure 2).

### Measurements of cortisol

**Figure 3:**
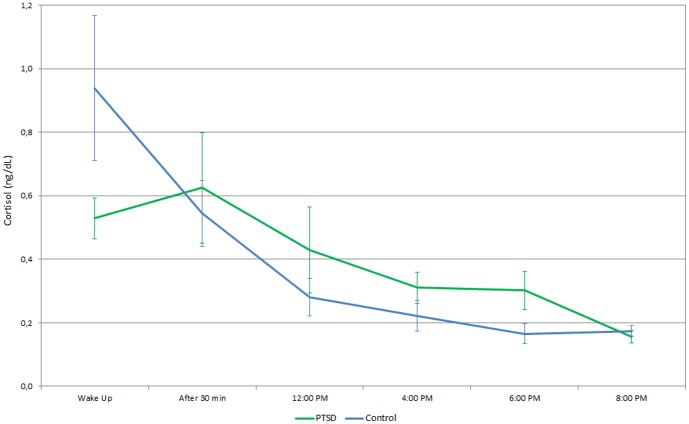
Cortisol. Distribution of mean cortisol values throughout the day in cases of PTSD and controls.

Of the 144 individuals initially selected for the study, 75 refused to take part, 18 missed evaluation appointments, and 3 presented technical difficulties in performing the test. Therefore, the prednisolone suppression test was only administered to 47 individuals (32 cases and 15 controls). Among the individuals who were excluded of the cortisol assessment, 49.5% had a diagnosis of PTSD, 69.3% were female, 52.5% were married, 71.3% were born in São Paulo, whose average age was of 38.03 (SE = 1.07). On the other hand, for those who were included in the evaluation of salivary cortisol, 65.6% had a diagnosis of PTSD, 68.8% were female, 50.5% were married, 65.6% were born in São Paulo, whose average age was 37.22 (SE = 2.24). There were no statistical differences between groups. Results showed alterations in cortisol levels over the time, which were significantly different between the two groups (Figure 3). The main difference was observed at waking up, and lower levels of salivary cortisol were observed in PTSD+ (x = 0.529, se = 0.006) than in PTSD- (x = 0.939, se = 0.228), and this difference was statistically significant (p<0.04). The data from post hoc analyses, comparing individual time points, showed difference only at 6:00 PM. The respective time points and corresponding p values were: wake up (p = 0.08); after 30 min (p = 0.62); 12:00 PM (p = 0.57); 4:00 PM (p = 0.19); 6:00 PM (p = 0.04) and 8:00 PM (p = 0.31).

## Discussion

The perception of having non-controlling mothers and caring fathers, during childhood and adolescence, was protective against the development of PTSD following exposure to severe urban violence in adulthood. The cluster analysis, using combined PBI scores for fathers and mothers, showed that individuals with low-control parents were less likely to develop PTSD. The evaluation of the neuroendocrine response, using the prednisolone suppression test, showed lower levels of salivary cortisol upon waking up and thirty minutes after awakening in the PTSD+ group. The PTSD+ group also appeared to have a smaller total area of the CC than PTSD- patients, but this difference was not statistically significant.

The role of parental relations in the development of mental disorders in adulthood has been demonstrated in a thirty-year follow up study conducted by Collishaw et al [25]. The authors concluded that the perception of parental care in childhood and the quality of affective bonds in adolescence and adulthood were predictive of resilience in adulthood. Studies have also found that parental bonds characterized by high care (affective bonding) and low control (incentive to autonomy), are protective against the development of psychiatric symptoms in adulthood, while bonds involving low care (neglectful bonds) and excessive control (intrusive bonds) represent risk factors [26].

The relevance of maternal bonding for healthy development is well-known in the literature. However, it is also important to investigate the importance of affectionate paternal behavior for individual and family dynamics, childhood development and later mental health. A study on 8441 participants, of the National Child Development study, explored the association between father involvement in childhood/adolescence and psychological distress in adulthood, while controlling for maternal involvement and other known confounds. The authors concluded that father involvement at age 7 was a protective factor against psychological maladjustment in adolescent individuals from non-intact families. The results of the study also showed that, in women, paternal involvement at age 16 was protective against psychological distress during adulthood [27].

Studies have shown that the effects of environmental adversity on mental health can be modulated by a number of genetic polymorphisms [28]–[30]. On the other hand, a recent review by Szyf [31] showed that early social adversity may lead to epigenetic alterations which could, in turn, lead to individual differences in the response to stress and in mental health over the course of life. The interaction between genetic polymorphisms and environmental factors may also contribute to the protective effect of parental care on mental health. When exposed to stress later in life, peer-reared macaques, who were separated from their mothers and had at least one short allele for the serotonin transporter gene (rh-5HHTLTR), exhibited higher rates of ACTH than non-separated animals. The same effect has not been observed in primates reared with their mothers, who present no differences in neuroendocrine response, regardless of the presence of genetic polymorphisms [32].

Possible explanations for the association between parental bonding, HPA axis response, and predisposition to PTSD in adulthood may be related to mechanisms of gene-environment interaction, as well as molecular mechanisms and their effects on the neuroendocrine response [33]. Further studies addressing the identification of susceptibility genes for PTSD, like polymorphisms in the dopamine transporter gene [34] and in the promoter region of the 5-HT transporter gene [35], would greatly increase the understanding of stress-related disorders and contribute to the comprehension of their molecular basis and distinct neuroendocrine signature in individuals who report early life stress (ELS). Thus, it is possible that episodes of ELS, by means of epigenetic mechanisms of glucocorticoid receptor sensitization, predispose the individual to HPA axis alterations, such as increased cortisol signaling capacity and sympathetic activation, which are common in individuals who suffer from PTSD [13]. These findings reinforce the idea that the blunted response to the prednisolone suppression test, observed in PTSD group, could be related to the decrease of basal cortisol levels and GR super-sensitivity, and reveal that the quality of parental bonding, during childhood and adolescence, could significantly influence HPA-axis reactivity through their impact on the stress response.

Noteworthy to mention that hypocortisolism in PTSD occurs in the context of increased HPA-axis sensitivity to negative glucocorticoid (GC) feedback [11], despite a marked and sustained increase of corticotrophin releasing factor (CRF) concentration in the cerebrospinal fluid (CSF) [36]. Evidence of a blunted ACTH response to CRF stimulation in PTSD supports the hypothesis that its pathology includes elevated levels of hypothalamic CRF activity and the consequent down-regulation of pituitary CRF receptors [11]. Together, this constellation of neuroendocrine findings in PTSD reflects the sensitization of the HPA axis, when these patients are exposed to stressors. The dexamethasone suppression test (DST) is used to evaluate GC negative feedback, particularly at the pituitary level [37]. Although greater cortisol and ACTH suppression after DST has been found in PTSD patients, this finding does not explain much about the contribution of higher brain areas to the activity of the HPA axis, because dexamethasone does not easily penetrate the blood-brain barrier; therefore, it does not bind to the mineralocorticoid receptor (MR) [38]. The prednisolone suppression test (PST) avoids this problem, because its pharmacological properties bear great similarity to endogenous cortisol, and its effect on the HPA axis is closer to natural physiological adaptations [21]. Previous studies have shown that 20 mg/day of prednisolone induces cortisol suppression [39]. Pariante and colleagues [21], who developed the PST, report that prednisolone suppresses salivary cortisol to a larger extent than plasma cortisol, and, compared to dexamethasone, plasma–salivary correlations are more consistent during a PST. They propose that administration of 5 mg prednisolone, which suppresses approximately 30% of salivary cortisol secretion, is the ideal tool to investigate.

Throughout the neurodevelopmental process, the CC plays an important role in interhemispheric connections and in the processing of emotional and mnemonic stimuli associated with parental bonding. Although no callosal differences were found in the present investigation, there have been studies showing reductions in the area or structural integrity of the posterior CC in cases of parental neglect [14] and childhood maltreatment [14]. Another issue that deserves mention is the high variability in callosal area in the PTSD- group (Figure 2). One possible explanation for this degree of variability is that, in general, larger samples are associated with lower variability, and the sample size of the PTSD+ group was twice that of the PTSD- group.

Since the inclusion of patients with some mental disorders, which are commonly co-morbid with PTSD, could lead to findings that are not generalizable to most PTSD populations, the present study excluded patients who presented with generalized anxiety disorder, panic disorder, obsessive compulsive disorder, and MDD, before the traumatic event that triggered the PTSD. Patients who were diagnosed with MDD or other disorders, concomitantly with or following the PTSD diagnosis, were not excluded. Patients with psychiatric disorders, but who had been symptom-free (remitted) for at least six months before the event that triggered the PTSD.

It is important to note that this was a case-control study, and reverse causality could not be discarded. Additionally, the sample sizes for the salivary cortisol tests and neuroimaging assessments were somewhat small, reducing the statistical power of between-group comparisons. Small samples sizes represent a significant limiting factor when interactions are tested. Since the interactions require greater statistical power, there are some concerns about interpreting results, particularly for cortisol and corpus callosum analyzes. Also, the lack of a more rigid control over cortisol sampling time (because subjects were at home) is a drawback for our results. We were not able to confirm exact time of wake-up, which made impossible to calculate area under the curve. Moreover, the subsamples used for each of these measurements (Corpus Callosum and cortisol) were largely distinct. All control subjects in the present study were victims of violence who did not develop PTSD, so that the present study may also have contributed to the study of risk and resilience factors for PTSD.

Nonetheless, the present results underscore the impact of healthy parental bonding, characterized by low control and high affection, their association with HPA axis response, and their role as a possible protective factor against the development of PTSD in adulthood. The understanding of risk factors associated with psychiatric morbidity in adulthood may greatly benefit from investigations of childhood parental bonding and of the interactions between early environment, neuroendocrine stress response and neurodevelopment. Future studies, with a longitudinal design and satisfactory length of follow-up, will be necessary to confirm the present findings and provide further information, regarding the effect of interactions between early parental bonding and genetic factors n on the neuroendocrine stress response profile. The further investigation of early parental bonds may also contribute to the understanding of risk and resiliency factors involved in the development of PTSD.
